# Gene expression and adaptive noncoding changes during human evolution

**DOI:** 10.1186/s12864-017-3831-2

**Published:** 2017-06-05

**Authors:** Courtney C. Babbitt, Ralph Haygood, William J. Nielsen, Gregory A. Wray

**Affiliations:** 1 Department of Biology, Duke University, Durham, NC 27708 USA; 2 Institute for Genome Sciences & Policy, Duke University, Durham, NC 27708 USA; 3 Ronin Institute, Montclair, NJ 07043 USA; 4 Department of Evolutionary Anthropology, Duke University, Durham, NC 27708 USA; 5 Present Address: Department of Biology, University of Massachusetts Amherst, Amherst, MA 01003 USA

**Keywords:** Adaptation, Gene expression, Gene function, Gene regulation, Human evolution

## Abstract

**Background:**

Despite evidence for adaptive changes in both gene expression and non-protein-coding, putatively regulatory regions of the genome during human evolution, the relationship between gene expression and adaptive changes in *cis*-regulatory regions remains unclear.

**Results:**

Here we present new measurements of gene expression in five tissues of humans and chimpanzees, and use them to assess this relationship. We then compare our results with previous studies of adaptive noncoding changes, analyzing correlations at the level of gene ontology groups, in order to gain statistical power to detect correlations.

**Conclusions:**

Consistent with previous studies, we find little correlation between gene expression and adaptive noncoding changes at the level of individual genes; however, we do find significant correlations at the level of biological function ontology groups. The types of function include processes regulated by specific transcription factors, responses to genetic or chemical perturbations, and differentiation of cell types within the immune system. Among functional categories co-enriched with both differential expression and noncoding adaptation, prominent themes include cancer, particularly epithelial cancers, and neural development and function.

**Electronic supplementary material:**

The online version of this article (doi:10.1186/s12864-017-3831-2) contains supplementary material, which is available to authorized users.

## Background

The evolutionary mechanisms responsible for divergence in gene expression between species are poorly understood. To begin with, expression level is for most genes a high-dimensional phenotype: almost without exception it differs among cell types, across the life cycle, and in response to numerous environmental factors [1, 2]. This makes it challenging to link positive selection on regulatory sequences to any particular aspect of a gene’s expression. In addition, populations often harbor significant levels of genetic variation that influences gene expression [3], confounding attempts to distinguish between-species divergence from within-species variation. Finally, the full complement of *cis*-regulatory elements is rarely known, constraining attempts to carry out comprehensive scans for natural selection. Perhaps unsurprisingly, it has proven difficult to detect a clear relationship at a genomic scale between the distribution of positive selection on noncoding sequences and divergence in gene expression, particularly in multicellular organisms [4].

The question is whether this result is a true or false negative. One way to move beyond a quest for simple correlations is to carry out joint analysis of genes that contribute to related phenotypes. During human origins, for instance, many of the same functional categories of genes show enrichments for signatures of positive selection [5–8] and for changes in tissue-specific expression level [9–14], even though on a gene-by-gene basis no correlation is evident. This overlap in enrichments hints at cause-and-effect or common-effect relationships. For example, two genes whose products are part of the same biological process might both experience positive selection to change expression levels because they both alter the same quantitative organismal trait in the same direction. The clearest examples come from metabolic pathways [15–17], but in principle this relationship could apply to any set of genes that contribute to the same trait or set of traits. Working with functionally related sets of genes rather than single genes should, in principle, improve our ability to detect relationships between signatures of positive selection and gene expression change.

To test this concept, we measured transcript abundance in five tissues in humans and chimpanzees, and then used the results to assess the relationship between gene expression and positive selection. We chose tissues informative to understanding the evolution of metabolism, namely adipose tissue, cerebellum, cortex, liver, and skeletal muscle. These tissues provide an opportunity to explore the “expensive tissue” hypothesis that a major shift in energy allocation among tissues occurred during human evolution in order to support the remarkable expansion of the metabolically expensive human brain [14, 18–21]. We analyzed our measurements of transcript abundance from all tissues together in a single statistical model so as to maximize our power to detect expression differences. We then looked at correlations between differential gene expression and the results from three noncoding DNA datasets. These included a combination of human accelerated regions (where there are significantly more changes on the human branch in a highly-conserved regions in other organisms, anywhere in the genome) [7, 8]; as well as rapidly evolving putative *cis*-regulatory regions upstream of coding sequences (as compared to changes in local introns) [6]. As expected, we find little correlation between gene expression changes and adaptive noncoding (putative *cis*-regulatory) changes at the level of individual genes; however, we did find appreciable correlations between the two for several informative kinds of biological functions. Our results demonstrate the utility of considering functional categories when studying the evolution of gene expression and provide novel insights into the genetic basis for human origins.

## Results

### Measurements of gene expression

**Fig. 1:**
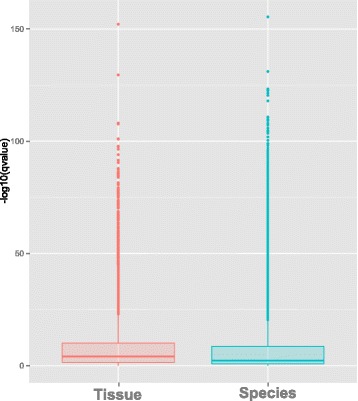
Boxplot of the –log10(qvalue) distributions from the GLM examining the Tissue (left, orange) and Species (right, green) effects

We used RNA-Seq to measure expression of 14,341 genic regions in at least one of white adipose tissue, lateral cerebellum, frontal cortex, liver, and skeletal muscle sampled from four male humans and four male chimpanzees (see Additional file 1: Table S1 for details). Using a multi-factor Generalized Linear Model (GLM) to analyze our measurements of all tissues together [22], we identified 4828 genes whose expression differs between humans and chimpanzees (q-value <0.05, 5% FDR). This proportion, 34% (4828 of 14,341), is greater than in previous studies that analyzed different tissues in separate models [9, 13, 23, 24]. GO analyses of overall differential expression show significant expression differences in categories related to cell signaling, neural processes, ion transport and development (Fig. 1). In keeping with many previous studies [25–27], cluster analysis revealed a greater overall similarity in expression by tissue than by species; the tissue term in our GLM on average explained a greater fraction of differential expression (Kolmogorov-Smirnov test, *D* = 0.16069, *p*-value <2.2e-16) than did the species term (Fig. 1).

### Changes in expression divergence and tissue specificity

**Fig. 2:**
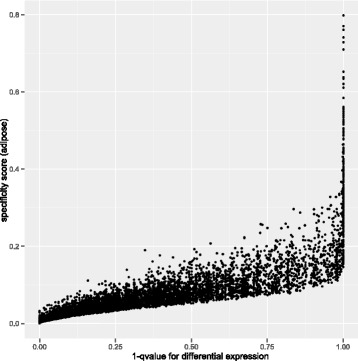
Correlation between tissue specificity and gene expression divergence between humans and chimpanzees. There is a significant correlation between higher specificity to a tissue and expression divergence (adipose tissue as an example here)

We then examined the trends of genes that have diverged in expression levels between humans and chimpanzees in individual tissues. We calculated a specificity score for differential expression (human vs. chimpanzee) in a given tissue relative to the rest of our gene expression data [as in 6, 28]. This score is not based on the magnitude of expression levels, but rather on the distribution of expression over tissues. A gene that is mainly differentially expressed in one tissue (even if at low numbers) between humans and chimpanzees will have a specificity score closer to one, and genes not showing changes in expression will have scores closer to 0. We found a strong correlation of tissue specificity and changing gene expression between humans and chimpanzees in all five tissues (adipose: rho = 0.962,liver: rho = 0.828, cerebellum: rho = 0.708, rho = cerebral cortex: 0.833, rho = skeletal muscle: 0.757; all 5 *p*-values <2.2e-16) (example tissue shown in Fig. 2). These results suggest that the more tissue specific a gene’s expression, the more likely its expression is to change over evolutionary time, even during the short divergence time between the human and chimpanzee lineages. In contrast, the categories of genes we found enriched for similar expression levels across the tissues we measured here are mainly comprised of housekeeping processes, such as “transcription regulation” and “DNA binding”. This is consistent with the idea that tissue-specific selection pressures are more easily accommodated by changes in cis-regulation than by changes in protein structure, because the latter are more likely to have deleterious pleiotropic effects [2, 29].

**Fig. 3:**
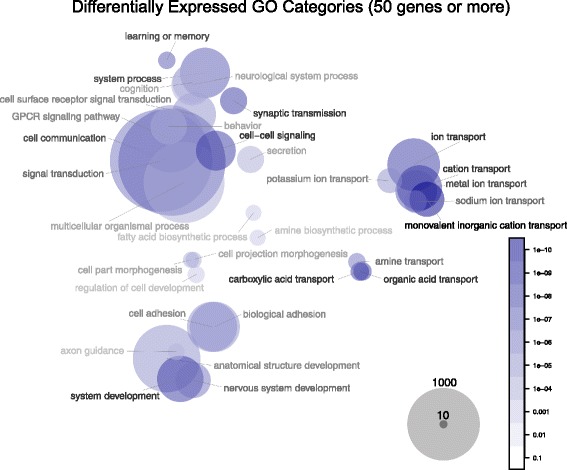
Bubble plot of GO Biological Process enrichments for gene expression differences across five tissues between human and chimpanzee. To display these traits visually, we calculated an optimal 2-dimensional arrangement using non-metric multidimensional scaling on the between-category sematic similarity scores, a measure of a priori relatedness of traits. Traits whose SimREL distance is less than 0.5 are considered similar within the GO Biological Process ontology tree (code available on request). The significantly differentially expressed categories are displayed on the same axes, but separated into two plots for clarity. In each plot, the intensity of the colors of the circles and text indicate the evidence for differential expression of each trait. The area of the circle is proportional to the log of the number of genes counted in each trait

**Fig. 4:**
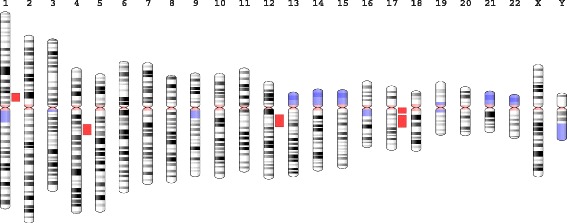
Ideogram of clustering of significant differentially expressed genes in humans compared to chimpanzees across tissues (red bars highlight the chromosomal enrichment regions in Table 1)

### Functions of differentially expressed genes across humans and non-human primates

Considering just the large (>50 genes) Gene Ontology categories (Fig. 3) we see themes of metabolism, signaling, development, and nervous system as differentially expressed between humans and chimpanzee (Fig. 3). These results are intriguing, but only represent biological processes functioning in normal tissue.

To further understand the implications of these expression differences, we analyzed their distribution over multiple gene sets using the Molecular Signatures Database (MSigDB) [30, 31]; see “Methods” for details). MSigDB consists of 10,348 sets grouped into 20 collections representing broad aspects of gene function and organization. The collections form a shallow, partially hierarchical, tree structure. One of the challenges in interrogating these large gene ontology sets is the redundancy in, and heterogeneous size of, these gene set ontologies [32]. For example, the C3:All collection is the union of the C3:MIR and C3:TFT collections with no deeper branching. Therefore, we considered only 16 leaf (of the tree structure) collections (e.g., C3:MIR and C3:TFT but not C3:All). In order to emphasize broad trends among changes in gene expression between humans and chimpanzees, we restricted attention to sets containing at least 50 genes whose expression we measured.

Table 1 lists the 20 (of 5247) MSigDB gene sets most enriched with genes scoring high for overall differential expression between humans and chimpanzees (see Additional file 2: Tables S2a–S2f for full results, both overall and per tissue). One of the most unexpected and striking patterns is the clustering of statistically differentially expressed genes at specific cytogenetic bands (Fig. 4, Table 1). Some of these regions have interesting evolutionary histories in primates, and have important links to human disease. There is a common inversion in chromosome 17q11.31 that segregates within human populations [33] and is correlated with a number of neurological conditions, such as Parkinson’s [34] and intellectual disabilities [35] and intercranial volume [36]. This region is also bounded by a segmental duplication that seems to have been under positive selection in the primate lineage [37], and contains genes such as MAPT and BRCA1. The 17q11 region is associated with a number of immune conditions, such as tuberculosis and leprosy in human populations [38], and has an expanded repertoire of cytokines [39], while the 12q13 region is associated with Type I diabetes [40]. Additionally, the 17q11 and 12q13 cytogenetic bands have gene clusters that have expanded during primate evolution [41]. There is previous evidence of positive selection in regions with duplications, both in coding [42] and in regulatory sequence [37], which we discuss below. Additionally, Table 1 shows that differentially expressed genes between humans and chimpanzee across all five tissues are enriched for processes related to neural signaling [43, 44] and pathways in cancer [45–47], based on the data from those studies.

**Table 1:** Functions of overall differentially expressed genes

Set	Collection	# genes	r_rb	SE(r_rb)
chr17q11	C1:All	66	0.88	0.021
chr12q13	C1:All	152	0.84	0.026
NIKOLSKY BREAST CANCER 17Q11 Q21 AMPLICON	C2:CGP	80	0.84	0.033
chr17q21	C1:All	164	0.69	0.033
chr4q21	C1:All	61	0.62	0.066
chr1p13	C1:All	79	0.59	0.06
GNF2 DNM1	C4:CGN	63	0.41	0.046
RICKMAN HEAD AND NECK CANCER A	C2:CGP	77	0.34	0.06
LEIN NEURON MARKERS	C2:CGP	50	0.34	0.052
CAHOY NEURONAL	C6:All	84	0.33	0.053
GCM MAP1B	C4:CGN	53	0.33	0.076
ANASTASSIOU CANCER MESENCHYMAL TRANSITION SIGNATURE	C2:CGP	53	0.33	0.069
SABATES COLORECTAL ADENOMA UP	C2:CGP	86	0.33	0.049
GNF2 CCNA2	C4:CGN	52	0.32	0.065
KRAS.KIDNEY UP.V1 UP	C6:All	113	0.32	0.047
VOLTAGE GATED CATION CHANNEL ACTIVITY	C5:MF	57	0.31	0.061
NAKAYAMA SOFT TISSUE TUMORS PCA2 UP	C2:CGP	71	0.31	0.053
GCM MAPK10	C4:CGN	71	0.31	0.059
CERVERA SDHB TARGETS 1 UP	C2:CGP	95	0.3	0.052
VOLTAGE GATED CHANNEL ACTIVITY	C5:MF	63	0.3	0.058

The 20 (of 5247) MSigDB gene sets most enriched with genes scoring high for overall differential expression between humans and chimpanzees are listed. Collection is the MSigDB collection containing the set (one of 16). genes is the number of genes whose expression we measured in the set (at least 50). *r*_rb_ is the rank-biserial correlation between scores for differential expression and membership in the set; sets are ordered by decreasing *r*_rb_. SE(*r*_rb_) is the standard error of *r*_rb_ via bootstrapping with 10,000 replicates

We then went on to examine whether the differentially expressed genes between humans and chimpanzees have roles in human disease or dysregulation. The KEGG pathways that show enrichments are clearly related to two general groups of processes: neuronal function and cancer (Table 1, Additional file 3: Table S3). There are a number of categories related to “neuronal signaling.” For processes related to cancer, the signal appears to be coming from differential gene expression in processes related to cell signaling and adhesion (e.g. “Focal adhesion”, “Gap Junction”, and “ECM Receptor Interaction”). This is suggestive that healthy differential gene expression between humans and chimpanzees overlaps with gene also involved in some late-onset disease susceptibilities.

### Genic correlations between differential expression and noncoding adaptation

Four thousand five hundred eighty-one genes whose expression we measured were also analyzed in our previous study of noncoding (putative *cis*-regulatory) regions immediately upstream from transcription start sites [6]. Consistent with that study, the correlation between our current scores for overall differential expression between humans and chimpanzees (q-value <0.05) and our previous scores for adaptive sequence changes during human evolution is negligible (rank correlation *r*_r_ = 0.0074, one-tailed *p* = 0.32 via permutation test with 10,000 permutations). The tissue with the highest correlation between scores for per-tissue differential expression and scores for adaptive sequence changes is cerebral cortex, but the correlation is weak (*r*_r_ = 0.031, *p* = 0.032). Similar analyses using scores for adaptive sequence changes from two other studies of noncoding regions [7, 8] yield similar results (see Additional file 4: Table S4, with total, and by tissue, tabs for full results). Thus, per gene, the relationship between gene expression and adaptive noncoding changes is apparently weak.

Beyond the possibility of noise or errors in the scores, this finding is unsurprising for several reasons explained above. We measured expression in only a few tissues from healthy adults, giving us a limited view of this high-dimensional expression phenotype. For these and other reasons, we had not expected to see a strong relationship between gene expression and adaptive noncoding changes at the level of individual genes.

### There are correlations between differential expression and adaptation at the level of ontology categories

However, we hypothesized that there might be a stronger relationship at the level of biological function than at the level of individual genes. Conceivably, both genes whose expression differs between humans and chimpanzees and genes near noncoding regions whose sequences changed adaptively during human evolution might tend to affect the same biological functions. Of course, biological function is an extremely broad notion. As the diversity of MSigDB collections attests, genes can be categorized in many different ways, from their molecular characteristics through their contributions to normal development and physiology to their involvement in pathologies such as cancer. Accordingly, our question amounts to whether there is some type of biological function with respect to which there is a stronger relationship between gene expression and adaptive noncoding changes. We addressed this question using the commonly accessed MSigDB gene ontology collections [30, 31].

For each MSigDB gene set delineated above and for each previous study of noncoding regions mentioned above [6–8], we computed a rank-biserial correlation (see Materials and Methods) of the kind presented in Table 1 for enrichment with genes scoring high for adaptive sequence changes in putatively regulatory regions of the human genome. As in our previous meta-analysis of such studies [5], we combined these rank-biserial correlations across the studies to obtain one rank-biserial correlation for each set, restricting attention to sets represented in at least two of the studies, with correlations not significantly heterogeneous across these studies, and containing at least 50 genes analyzed on the average over these studies (see “Methods” for details). For each MSigDB collection delineated above, we then computed the correlation over gene sets in the collection between enrichment rank-biserial correlations for differential expression, either overall or per-tissue, and enrichment rank-biserial correlations for noncoding adaptation. Interpreting these correlations is complicated by overlap among sets within a collection—a typical gene is a member of several sets—which tends to inflate the correlations. Thus, for each correlation, we corrected by computing a *p*-value using a form of permutation testing that accounts for overlap among sets.

Table 2 lists the results for overall differential expression (see Additional file 5: Tables S5b–S5f for per-tissue results). The most significant correlations represent several types of function with respect to which there is appreciable congruity between gene expression and adaptive noncoding changes. The C3:TFT collection contains sets of genes with binding sites for a transcription factor according to the TRANSFAC database. The sets most co-enriched with both overall differential expression and noncoding adaptation include targets of *FOXM1*, *POU3F1* and *POU3F2*, *GATA1*, and *NKX2–5*, which are regulators of the cell cycle, neuron development, erythrocyte development, and cardiomyocyte development, respectively [48–51]. The C2:CGP collection contains sets of genes whose regulation is altered in response to a genetic or chemical perturbation. The most co-enriched sets include MOHANKUMAR_TLX1_TARGETS_DN [52], genes down-regulated by TLX1 in a breast cancer cell line; POOLA_INVASIVE_BREAST_CANCER_UP [53], genes up-regulated in tissues from patients with vs. without breast cancer. Beyond these three collections, the most co-enriched sets include C4:CGN:MORF_THPO and MORF_EPHA7 [54], genes in the expression neighborhoods of the cancer-associated genes THPO and EPHA7, respectively; and C5:BP:SYNAPTIC_TRANSMISSION and NERVOUS_SYSTEM_DEVELOPMENT, genes involved in these biological processes according to the GO database [55]. There are prominent themes here, notably cancer and neural development and function.

**Table 2:** Functional correlations between overall differential expression and noncoding adaptation

Collection #	sets	r_r	p(r_r)	q(r_r)
C3:TFT (transcription factor targets)	472	0.35	< 0.0001	< 0.0005
C2:CGP (chemical and genetic perturbations)	645	0.28	< 0.0001	< 0.0005
C7:All (immunologic signatures)	1723	0.14	< 0.0001	< 0.0005
C4:CGN (cancer gene neighborhoods)	88	0.5	0.012	0.045
C6:All (oncogenic signatures)	109	0.22	0.019	0.057
C4:CM (cancer modules)	87	0.29	0.036	0.09
C5:CC (GO cellular components)	37	0.33	0.14	0.27
C3:MIR (microRNA targets)	109	0.11	0.14	0.27
C1:All (positional gene sets)	9	0.17	0.34	0.53
H:All (hallmark gene sets)	25	0.089	0.35	0.53
C2:CP:Reactome (Reactome gene sets)	39	0.058	0.4	0.55
C2:CP:KEGG (KEGG gene sets)	11	−0.17	0.7	0.88
C2:CP:Other (other canonical pathways)	6	−0.49	0.8	0.93
C5:BP (GO biological processes)	123	−0.28	0.95	0.96
C5:MF (GO molecular functions)	44	−0.41	0.96	0.96

All MSigDB collections we considered are listed except for C2:CP:BioCarta, in which no gene set contained at least 50 genes analyzed in a previous study of adaptive noncoding changes. # sets is the number of gene sets we considered in the collection. *r*_r_ is the rank correlation between rank-biserial correlations for enrichment with differential expression (each of which is a rank-biserial correlation as in Table 1) and rank-biserial correlations for enrichment with noncoding adaptation (each of which is likewise a rank-biserial correlation). *p*(*r*_r_) is the one-tailed *p*-value of *r*_r_ via permutation test with 10,000 permutations; collections are ordered by increasing *p*(*r*_r_), which reflects *r*_r_, # sets, and patterns of overlap among gene sets in the collection

## Discussion

The correlations that we detected between positive selection and changes in gene expression are significant, but not strong. There are several plausible reasons for this, beyond whatever noise exists in the expression measurements and selection scores. It is possible, for instance, that some expression differentiation is neutral or even deleterious, and that some of the adaptation happened along the chimpanzee rather than human lineage. It is also important to bear in mind that both datasets contain only a subset of all possible genes and tissue types over the four individuals. Additionally, several recent studies have shown the importance of looking over multiple tissue types to gain a clearer understanding of important *cis*-regulatory effects within human populations, where eQTLs differ among or across environments [56–58]. Including more expression data would also likely improve the correlation between expression differences and the scans for selection in our study as well as in other, more distal putative enhancer regions [7, 8].

Nevertheless, there are other studies that support the idea that one can detect the effects of adaptation on *cis*-regulation on human traits. A correlation between expression divergence in other tissues and mutations in short core promoter regions has been reported [12]. More recently, an analysis of positive selection across the human genome using human population genomic data (1000 Genomes Project [59]) found that signatures of adaptation are common in regulatory regions (as defined by open chromatin) [60]. These are signals of more recent positive selection than were detected in Haygood et al. [[6]], suggesting that these regulatory regions are continuing to adapt to novel challenges and environments, and, presumably, this correlates with changes in gene expression.

The enrichments for differential expression between humans and chimpanzees by cytogenetic band was somewhat unexpected. For example, the category C4:CGN (cancer gene neighborhoods, q-value = 0.045, Table 2), shows up as significant in our correlations between noncoding adaptation and differential expression. It is possible there are more global regulators of expression for these regions that differ between species. Compared to the genomic regions highlighted in [37, 42, 61], there does seem to be an overlap between these regions and regions where segmental duplications have occurred in the human genome. This could influence some of our signal of positive selection due to orthology issues; however, there is previous evidence of positive selection in regions with duplications, both in coding [42, 61] and in regulatory sequence [37].

We also found a strong correlation between tissue specificity and shifting gene expression between species. This makes sense in the light of pleiotropic concerns, where genes with a greater degree of tissue specificity can change expression pattern or levels over relatively short evolutionary time spans.

One important question is whether there are pleiotropic consequences of these adaptive changes in gene expression that have accrued during human evolution. We find that disease pathways involved in signaling and certain forms of cancer are enriched in the genes that show differential expression between humans and chimpanzees. There appears to be a substantial difference in the frequency of epithelial derived cancers, such as breast, ovary, and prostate between humans and non-human primates: incidence of cancer is significantly higher in humans compared to non-human primates in captivity [62–65], reviewed in [66] and human fibroblasts also show reduced apoptotic ability as compared to chimpanzees and other non-human primates [67]. Although much of this difference is likely driven by environmental and dietary factors, some of the difference may be due to genomic differences between the species. It will take functional studies to understand how these gene expression differences are driving the differences in organismal phenotypes between humans and our closest relatives, and to understand how much of the differences in gene expression we see are due to genetic as opposed to environmental factors.

## Conclusions

We find that adaptation in regulatory regions may have driven many of these changes in gene expression, but that may have also had other attendant consequences related to human disease states. In looking at changes across tissues and evolutionary time, we see that shifting specificities across tissues show how gene expression can be the raw material for selection. These data present a window into how positive selection has worked to change gene expression between humans and chimpanzees at the level of larger groups of changing gene expression. These changes underlie the more obvious changes in phenotype, but may have the important side effect of also underling differential disease susceptibilities that may not have been visible to selection.

## Methods

### Sample preparation and sequencing

We obtained samples of white adipose tissue, lateral cerebellum, frontal cortex, liver, and skeletal muscle from four male humans and four male chimpanzees. The small sample size is due to the limited resources of chimpanzee post-mortem tissues, all from the same individual. Our experimental design was to match the number of individuals across the two species. All samples were obtained through opportunistic sampling of individuals that died of causes unrelated to this research. We obtained human samples from BioChain (Newark, CA) and the NICHD Brain and Tissue Bank for Developmental Disorders and non-human primate samples from the Southwest Foundation for Biomedical Research, and the New Iberia Research Center. We matched samples from each individual across tissues, so that all samples labeled, for example, “human 1” represent the same *trans* environment.

All brain samples from non-human primates were dissected by the same researcher in order to maintain consistency. We stored samples at −80 °C. For more information, see Additional file 1: Table S1.

We isolated total RNA from the samples using RNeasy kits (Qiagen, Valencia, CA), including a DNase I treatment step. Ten micrograms of RNA became the starting material for an RNA-Seq library per sample. We constructed libraries using SOLiD Total RNA-Seq kits (Ambion, Austin, TX), which yield strand-specific reads. Libraries were sequenced at the Duke University Sequencing and Genomic Technologies Shared Resource (http://genome.duke.edu/cores-and-services/sequencing-and-genomic-technologies/). Sequencing yielded approximately 70 million 50–35 bp paired-end sequences per library.

### Sequence mapping and count analysis

We mapped sequences into the human and chimpanzee genomes (hg19, panTro3) using Tophat version 1.4.1 [68]. We constructed orthologous gene models using the Primate Orthologous Exon Database, version 2 (http://precedings.nature.com/documents/7054/version/1). We filtered them using Ensembl (http://useast.ensembl.org/index.html), removing homology types one2many and many2many while retaining homology types one2one and apparentone2one (human-chimpanzee). We also removed ribosomal genes in the RPL, RPS, MRPL, and MRPS families, which include many paralogs with unclear homologies.

We derived counts per gene using HT-Seq [69]. We analyzed them using edgeR [70], defining a multi-factor Generalized Linear Model (GLM) with tissue, species, and species-within-tissue-interaction effects [22]. As a score for differential expression of a gene, we used the negative of the natural logarithm of the adjusted *p*-value for the appropriate contrast of the GLM. The positional information in Fig. 4 was plotted using the Genome Decoration Page at NCBI.

### Tissue specificity analysis

We defined the specificity score of the gene for a tissue as the square of the cosine of the angle between the vector and the axis corresponding to the tissue [as in 6, 28]. This measure depends on the distribution of expression over tissues, but not the overall magnitude of expression. A highly specific gene to one tissue has specificity scores near 1 for this tissue and near 0 for others, whereas housekeeping genes, for example, might be highly expressed in all tissues, and have specificity values around 0.

### Analyses of gene ontology groups and comparisons between studies of adaptation and gene expression

We used the Molecular Signatures Database (MSigDB) version 5.0 [30, 31]. More specifically, we used the 15 leaf collections in the tree of all 20 MSigDB collections, with one addition. One MSigDB collection, the C2:CP (canonical pathways) collection, is neither a leaf nor strictly an internal node of the branching pathways, because it includes not only the union of the C2:CP:BioCarta, C2:CP:KEGG, and C2:CP:Reactome collections but also 253 other gene sets. Therefore, we treated these 253 sets as an additional leaf collection, the C2:CP:Other collection.

As a rank-biserial correlation for enrichment of a gene set with genes scoring high for differential expression, either overall or per-tissue, we used the rank-biserial correlation, *r*_rb_, over genes whose expression we measured between scores for differential expression and membership in the set. *R*_rb_ measures association between an ordinal variable and a dichotomous variable [71]. It is proportional to the standard (Pearson) correlation between the ranks of the ordinal variable and any two values, say, 0 and 1, for the dichotomous variable. It is a linear function of the Mann-Whitney *U* statistic, and a test of *r*_rb_ = 0 is equivalent to a Mann-Whitney test, which was used to test for categorical enrichment in several previous studies of adaptive sequence changes [6, 28, 72, 73]. An important advantage of *r*_rb_ for our purposes is that there are well established procedures for combining it across studies [74]. We estimated the standard error of *r*_rb_, SE(*r*_rb_), as the standard deviation of *r*_rb_ over 10,000 bootstrap replicates.

Similarly, as a rank-biserial correlation for enrichment of a gene set with genes scoring high for adaptive sequence changes according to any one of three previous studies of noncoding regions [6–8], we used the rank-biserial correlation between scores for adaptive sequence changes and membership in the set. As in standard fixed-effects meta-analyses [74], we combined *r*_rb_ across studies to obtain its weighted mean, WM(*r*_rb_) = ∑_*i*_ *w*_*i*_ (*r*_rb_)_*i*_, where *w*_*i*_ = (1/SE(*r*_rb_)_*i*_)^2^ / ∑_*j*_ (1/SE(*r*_rb_)_*j*_)^2^. Under the null hypothesis (*r*_rb_)_*i*_ = (*r*_rb_)_*j*_ for every *i* and *j* from 1 to *n*, the heterogeneity statistic *Q* = ∑_*i*_ *w*_*i*_ ((*r*_rb_)_*i*_ − WM(*r*_rb_))^2^ has approximately the chi-squared distribution with *n* − 1 degrees of freedom, where the set is represented in *n* studies. We restricted attention to sets with *n* ≥ 2 and *Q* not significantly different from 0 (χ^2^_*n*−1_ *p* > 0.05). All code is available upon request.

### Functional correlations between noncoding adaptation and gene expression

The data for noncoding adaptation (Additional file 4: Table S4) are from Haygood et al. [5]. That was a meta-anaylsis of adaptation in coding and noncoding DNA. The three noncoding DNA datasets included a combination of human accelerated regions (where there are significantly more changes on the human branch in a highly-conserved regions in other organisms, anywhere in the genome) [7, 8] and rapidly evolving putative *cis*-regulatory regions near coding sequence (as compared to local introns) [6]. In each survey, we mapped the regulatory regions to the nearest genes with UniProt [75] identifiers and then to MSigDB categories. For each category, we computed the rank-biserial correlation, rrb, between the score for positive selection and membership in the category.

What we term the functional correlation between differential expression, either overall or per-tissue, and noncoding adaptation for an MSigDB collection is the rank (Spearman) correlation, *r*_r_, over gene sets in the collection; this is a correlation between enrichment rank-biserial correlations for differential expression (5% FDR q-value <0.05) and enrichment rank-biserial correlations for noncoding adaptation, restricting attention to sets containing at least 50 genes whose expression we measured. Additionally, noncoding categories have to be represented in at least two of the three previous studies of noncoding regions, with rank-biserial correlations for adaptation not significantly heterogeneous across these studies, and containing at least 50 genes analyzed on the average over these studies.

Gene sets overlap, so the enrichment rank-biserial correlations of different sets are not necessarily independent, which tends to inflate the correlations. Standard permutation testing, permuting rank-biserial correlations among sets within a collection, would be inappropriate. Therefore, instead, we permuted scores for differential expression and for adaptive sequence changes among genes, and for each permutation, we recomputed every enrichment rank-biserial correlation. These rank-biserial correlations represent a null hypothesis of no association between differential expression or adaptive sequence changes and membership in any gene set. Nonetheless, for any given permutation, they may exhibit correlations, not only by chance but also due to overlap among sets, which is preserved in their construction. For each collection, we estimated the one-tailed *p*-value of *r*_r_, *p*(*r*_r_), as the fraction of 10,000 permutation replicates in which the replicate *r*_r_ for the collection was no less than the observed *r*_r_ for the collection or as <0.0001 if there were no such replicates. In Table 2 (and Additional file 5: Tables S5a–S5f), we list not only *p*-values but also q-values, which adjust for multiple comparisons [76], although we are performing correlations on already adjusted q-values.

## Additional files

Additional file 1: Table S1. Details of individuals and samples. (XLSX 36 kb)

Additional file 2: Table S2. Functions of genes expressed differentially overall MSigDB gene sets. These sets are listed from most to least enriched with genes scoring high for differential expression between humans and chimpanzees overall. Collection is the MSigDB collection containing the set (one of 16). # genes is the number of genes whose expression we measured in the set (at least 50). rrb is the rank-biserial correlation between scores for differential expression and membership in the set; sets are ordered by decreasing rrb. SE(rrb) is the standard error of rrb via bootstrapping with 10,000 replicates. (XLSX 1260 kb)

Additional file 3: Table S3. A list of KEGG functions of overall differentially expressed genes. This list is restricted to KEGG pathways (i.e., MSigDB’s C2:CP:KEGG collection) and restricted to sets containing at least 50 genes. (XLSX 13 kb)

Additional file 4: Table S4. Genic correlations between differential expression and noncoding adaptation. Correlations are over genes whose expression we measured that were also analyzed in the studies of noncoding regions by Haygood et al. [6], Pollard et al. [7], and Prabhakar et al. [8]. # genes is the number of genes with appropriate scores. rr is the rank correlation between scores for differential expression between humans and chimpanzees, either overall or per-tissue, and scores for adaptive sequence changes during human or chimpanzee evolution, according to a study of noncoding regions. p(rr) is the one-tailed *p*-value of rr via permutation test with 10,000 permutations. (XLSX 49 kb)

Additional file 5: Table S5. Functional correlations between differential expression overall and noncoding adaptation. All MSigDB collections we considered are listed except for C2:CP:BioCarta, in which no gene set contained at least 50 genes analyzed in a previous study of adaptive noncoding changes. # sets is the number of gene sets we considered in the collection. rr is the rank correlation between rank-biserial correlations for enrichment with differential expression and rank-biserial correlations for enrichment with noncoding adaptation. p(rr) is the one-tailed *p*-value of rr via permutation test with 10,000 permutations; collections are ordered by increasing p(rr), which reflects rr, # sets, and patterns of overlap among gene sets in the collection. (XLSX 63 kb)

## Abbreviations

FDR: False discovery rate
GLM: Generalized linear model
MSigDB: Molecular signatures database
